# Bulgarian Translation, Cross-Cultural Adaptation, and Validation of the Simple Shoulder Test and the American Shoulder and Elbow Surgeons Shoulder Assessment Form

**DOI:** 10.7759/cureus.106313

**Published:** 2026-04-02

**Authors:** Nikolay Cherkezov, Aleksandar Iliev, Daniela Kovacheva-Predovska, Nikolay Dimitrov

**Affiliations:** 1 Orthopaedics and Traumatology, Specialized Orthopaedic University Hospital “Prof. B. Boychev”, Sofia, BGR; 2 Orthopaedics and Traumatology, Medical University - Sofia, Sofia, BGR; 3 Orthopaedics and Traumatology, University Hospital “St. Anna” - Sofia, Sofia, BGR; 4 Physical Medicine and Rehabilitation, University Hospital “St. Anna” - Sofia, Sofia, BGR; 5 Physical Medicine and Rehabilitation, Medical University - Sofia, Sofia, BGR

**Keywords:** bulgarian, prom, shoulder pain assessment, translation, validation

## Abstract

Background: Patient-reported outcome measures (PROMs) are essential for evaluating functional impairment, treatment effectiveness, and health-related quality of life in patients with musculoskeletal disorders. Among shoulder-specific PROMs, the Simple Shoulder Test (SST) and the American Shoulder and Elbow Surgeons Shoulder Assessment Form (ASES) are widely used instruments with established measurement properties. However, no validated shoulder-specific questionnaire has previously been available in the Bulgarian language.

Objective: To translate and culturally adapt the SST and ASES questionnaires into Bulgarian and evaluate their reliability, internal consistency, measurement error, convergent validity, and discriminant validity in patients with shoulder disorders.

Methods: Translation and cross-cultural adaptation were conducted following established forward-backward translation guidelines, expert committee review, and pilot testing. A total of 103 patients with shoulder pathology and 42 asymptomatic individuals were included. Construct validity was assessed by examining correlations between the Bulgarian SST (BSST) and Bulgarian ASES (BASES). Test-retest reliability was evaluated using intraclass correlation coefficients (ICC) with a 14-day interval. Internal consistency was determined using Cronbach’s alpha. Discriminant validity, floor and ceiling effects, and measurement error were also analyzed.

Results: Strong convergent validity was demonstrated between BSST and BASES (Pearson r = 0.719, p < 0.0001). Test-retest reliability was excellent for both instruments (ICC = 0.96 for BSST and 0.93 for BASES). Internal consistency was acceptable for BSST (Cronbach’s α = 0.77) and good for BASES (Cronbach’s α = 0.86). No significant floor or ceiling effects were observed. Both questionnaires successfully discriminated between patients with shoulder disorders and healthy controls (p < 0.0001).

Conclusion: The Bulgarian versions of the SST and ASES demonstrate good reliability and convergent validity and are suitable for assessing shoulder function and pain in Bulgarian-speaking populations. Their availability facilitates clinical evaluation, outcome monitoring, and participation in international research involving shoulder disorders.

## Introduction

Shoulder pain represents a frequent reason for consultation in primary care, with the yearly incidence of shoulder disorders estimated at approximately 7% [1]. Because of its susceptibility to degenerative processes-often related to acute injury or repetitive overuse-the glenohumeral joint is commonly affected, with osteoarthritis reported in 17-19% of individuals over the age of 40 [2]. Persistent shoulder pain and functional impairment substantially reduce quality of life, causing both physical and psychological burden, restricting everyday and work-related activities in younger populations, and compromising functional independence among older adults.

Evaluation of orthopaedic disease severity and treatment effectiveness has evolved considerably in recent decades. Increasing emphasis has been placed on the patient’s viewpoint as a key indicator of healthcare quality. This has led to the widespread adoption of patient-reported outcome measures (PROMs), which reflect this paradigm shift [3, 4]. PROMs are validated, standardized instruments designed to capture patients’ self-reported symptoms, functional status, quality of life, and perceived treatment results.

PROMs are commonly classified as either generic or condition-specific instruments. Generic measures are intended to assess health-related quality of life (HRQoL) across diverse diseases, patient groups, and clinical contexts. Although useful for broad comparisons, such tools often lack the responsiveness required to identify subtle but clinically important changes associated with a particular disorder or anatomical site. Consequently, condition-specific PROMs have been developed to address these limitations. For assessment of shoulder-related disorders, frequently used instruments include the American Shoulder and Elbow Surgeons (ASES) Shoulder Assessment Form, the Simple Shoulder Test (SST), the Constant-Murley Score, and the Disabilities of the Arm, Shoulder, and Hand (DASH) questionnaire [4-7].

For a health-related quality of life questionnaire to be applied in a different linguistic, cultural, or national context, a structured cross-cultural adaptation process is required to ensure equivalence with the original instrument. This process involves not only precise linguistic translation but also appropriate cultural adaptation of the questionnaire items. The goal is to maintain the instrument’s psychometric integrity, including its validity and reliability, in line with the widely accepted cross-cultural adaptation guidelines proposed by Beaton et al. [8].

The Simple Shoulder Test (SST) is a patient-reported questionnaire introduced by Matsen et al. in 1993 with the aim of minimizing respondent burden while assessing shoulder function in the context of everyday activities [6, 9]. The instrument comprises 12 binary (yes/no) items, each addressing the patient’s ability to perform a specific functional task. Scores are summed to produce a total ranging from 0, indicating severe functional limitation, to 12, reflecting normal shoulder function. The SST has been shown to be a valid, reliable, and responsive outcome measure and has been successfully translated and validated across multiple languages, including Arabic, Brazilian Portuguese, Persian, Italian, Japanese, Thai, and others [6, 7, 10]. Furthermore, the SST has demonstrated good discriminative capacity between asymptomatic individuals and patients with various shoulder disorders, such as osteoarthritis, rheumatoid arthritis, rotator cuff pathology, and adhesive capsulitis [4].

The ASES score was developed by the American Shoulder and Elbow Surgeons Society with the objective of standardizing outcome assessment and facilitating multicenter research in shoulder and elbow disorders [4, 11]. The instrument includes both a clinician-reported section and a patient-reported section; however, in routine clinical use, scoring is typically based solely on the patient-reported components, which consist of a visual analog scale (VAS) for pain and ten functional items. The overall score ranges from 0 to 100 points and is equally weighted between pain and function, each contributing 50 points. Pain is calculated by subtracting the VAS pain score from 10 and multiplying the result by 5. Functional status is assessed using ten items scored from 0 to 3, producing a maximum raw score of 30, which is subsequently converted to a 50-point scale by multiplication with a factor of 5/3. The pain and function subscores are combined to yield the final ASES score [4]. The ASES has demonstrated strong psychometric performance, including high validity, reliability, and responsiveness, across a range of shoulder conditions such as glenohumeral osteoarthritis, rotator cuff arthropathy, and shoulder arthroplasty [12].

At present, the only shoulder-related outcome measure with a validated Bulgarian version is the Disabilities of the Arm, Shoulder and Hand (DASH) questionnaire [13]. In comparison with the SST and ASES, the DASH includes a larger number of detailed items and requires a longer time for completion. Moreover, as a region-specific instrument intended to assess the entire upper limb, it may be less responsive to subtle or joint-specific functional changes in cases of isolated shoulder pathology. The need for an appropriate shoulder-specific assessment tool in the Bulgarian language is therefore evident, as no such validated instrument is currently available. Accordingly, the objective of this study was to translate and culturally adapt Bulgarian versions of the SST (BSST) and ASES (BASES), and to assess their reliability and validity in patients with shoulder disorders.

## Materials and methods

A cross-sectional and prospective cohort design was used to translate and culturally adapt the SST and ASES into the Bulgarian language and to evaluate their validity and reliability in patients with shoulder disorders.

Patients presenting with shoulder-related complaints were invited to participate in the study. Eligibility criteria included: (1) age ≥ 18 years, (2) native proficiency in Bulgarian, (3) literacy in Bulgarian, and (4) a shoulder condition diagnosed by an orthopaedic surgeon with a minimum symptom duration of one month. Exclusion criteria comprised a history of recent surgery or fracture of the affected shoulder, as well as the presence of neurological or musculoskeletal conditions unrelated to the shoulder pathology.

A total of 114 patients meeting the inclusion criteria were recruited from the outpatient clinic of University Hospital “St. Anna” - Sofia, Bulgaria. Participants were instructed not to initiate new treatments or significantly modify their activity levels during the 14-day interval between assessments. Nine participants did not complete the retest assessment, and two received therapeutic interventions prior to retesting; therefore, data from 103 patients (46 women and 57 men; mean age 54.2 ± 11.8 years; range 18-77 years) were included in the final analysis. In addition, 42 asymptomatic volunteers were enrolled to assess discriminant validity. Eighty seven patients were right hand-dominant and the right shoulder was affected in 69 patients. The most common reason for shoulder pain was tendinopathy, in 52 patients. Demographic data and patient characteristics are shown in Table 1.

**Table 1:** Demographic data and patient characteristics (n = 103) SD: standard deviation

Patients	N%
Sex	
Female	46 (44.7)
Male	57 (55.3)
Age	
Mean ± SD	54,2 ± 11,8
Dominant hand	
Right hand dominant	87 (84.5)
Left hand dominant	16 (15.5)
Affected right	69 (66.9)
Affected left	34 (33.1)
Diagnosis	
Bursitis	14 (13.6)
Rotator cuff tendinopathy	52 (50.5)
AC joint dislocation	8 (7.7)
Adhesive capsulitis	12 (11.7)
Rupture of rotator cuff	8 (7.8)
Shoulder instability	9 (8.7)
Education	
Middle school	21 (20.4)
High school	38 (36.9)
Bachelor’s degree or above	44 (42.7)

Procedure

Translation and Cross-Cultural Adaptation (Cross-Sectional Study)

The translation and cross-cultural adaptation of the ASES and SST were carried out in accordance with the well-established guidelines described by Beaton et al., after obtaining formal authorization from the original developers and copyright holders of both instruments [8]. The forward translation phase involved two native Bulgarian speakers: one professional linguist without medical training and one orthopaedic surgeon familiar with shoulder disorders and the underlying concepts of the questionnaires. These independent forward translations were subsequently compared and merged into a single synthesized version (T-12) through discussion and consensus.

The synthesized Bulgarian versions were then translated back into English by two independent translators, both native Bulgarian speakers residing in the United Kingdom with British citizenship and no medical background. An expert review committee - comprising the translators, members of the research team, and a physiotherapist specialized in orthopaedic rehabilitation - examined all versions to ensure semantic, idiomatic, experiential, and conceptual equivalence. Based on this review, pre-final Bulgarian versions of the ASES and SST were produced.

The pre-final questionnaires were pilot-tested in a sample of 30 patients with shoulder disorders to identify any linguistic or cultural inconsistencies. Following evaluation of patient feedback and minor adjustments where necessary, the final Bulgarian versions of the SST and ASES were established and subsequently used for psychometric validation (see Appendix A and Appendix B).

Psychometric Evaluation (Prospective Cohort Design)

Construct convergent validity was evaluated by having all participants complete both BSST and BASES questionnaires, which were administered in a randomized sequence to minimize potential order effects. To assess test-retest reliability, participants were instructed to complete the same instruments again after a 14-day interval. In addition, discriminant validity was examined by administering the BASES and BSST to a control group of 42 asymptomatic individuals.

Statistical Analysis

Statistical analyses were conducted using IBM SPSS Statistics for Windows, version 25 (IBM Corp., Armonk, NY, USA). Psychometric evaluation was performed in accordance with the COSMIN (COnsensus-based Standards for the selection of health Measurement INstruments) methodological framework for studies on patient-reported outcome measures, as described by Prinsen et al. [14]. Statistical significance was set at p < 0.05. Internal consistency was evaluated using Cronbach’s alpha coefficient, with values between 0.70 and 0.95 considered acceptable; coefficients below 0.70 were interpreted as indicating insufficient inter-item correlation, while values exceeding 0.95 suggested potential item redundancy, in line with the criteria proposed by Terwee et al. [15].

Construct (convergent) validity was examined using Pearson’s correlation coefficient to assess the association between BASES and BSST. Correlation coefficients were interpreted as excellent (0.81-1.00), very good (0.61-0.80), good (0.41-0.60), fair (0.21-0.40), or poor (0.00-0.20). A priori, a correlation coefficient of at least 0.70 between the BSST and BASES was hypothesized as evidence of adequate convergent validity.

Test-retest reliability (reproducibility) was assessed using intraclass correlation coefficients (ICC), based on data from the 103 patients who completed both questionnaires a second time after a 14-day interval. ICC values were interpreted as excellent (> 0.90), good (0.75-0.89), or moderate (0.50-0.74). Discriminant validity was evaluated by comparing BSST and BASES scores between patients with shoulder disorders and healthy controls using independent-samples t-tests.

Construct validity and responsiveness were further examined through the analysis of floor and ceiling effects. A ceiling effect was defined as scores between 90% and 100% of the maximum possible value, while a floor effect was defined as scores between 0% and 10% of the minimum possible value. The presence of floor or ceiling effects was assumed if more than 15% of participants achieved scores at either extreme. Measurement error was assessed by calculating the standard error of measurement (SEM) and the smallest detectable change (SDC) at the 95% confidence level (SDC₉₅) for both instruments. SEM was derived using the formula SEM = SD x √(1 - ICC), where SD represents the standard deviation of baseline scores and ICC the test-retest reliability coefficient. The SDC₉₅ was calculated as SDC₉₅ = SEM x 1.96 x √2, representing the smallest change that exceeds measurement error and reflects a true change in patient status [15]. In accordance with COSMIN guidelines, which recommend at least 50 participants for reliability and internal consistency analysis, our study included a total of 103 patients to ensure adequate statistical power and to account for potential missing data [14].

## Results

Translation and Cross-Cultural Adaptation

BSST and BASES were produced through a structured process involving forward and backward translation, pilot testing, and cultural adaptation, during which several modifications were introduced. The translation of the ASES questionnaire was completed without major challenges, with measurement units adapted to the metric system by converting pounds (lb) to kilograms (kg). In contrast, the cross-cultural adaptation of the SST required more extensive modifications. Unit conversions were necessary, including the transformation of pounds to kilograms and yards to meters. To enhance clarity and cultural relevance, several items were adapted, such as replacing a full pint container with a half-litre bottle and substituting a full gallon container with two 2-litre bottles. Distances of ten and twenty yards were converted to 9.5 and 18.29 meters, respectively, and subsequently rounded to 10 and 20 meters. Additionally, due to the limited familiarity with softball in Bulgaria, the softball referenced in question 9 was replaced with a tennis ball. During pilot testing in 30 patients with shoulder disorders, all participants reported clear understanding of the questionnaire items and instructions, and only minor refinements were required prior to finalization.

Validity and Floor/Ceiling Effects

**Figure 1:**
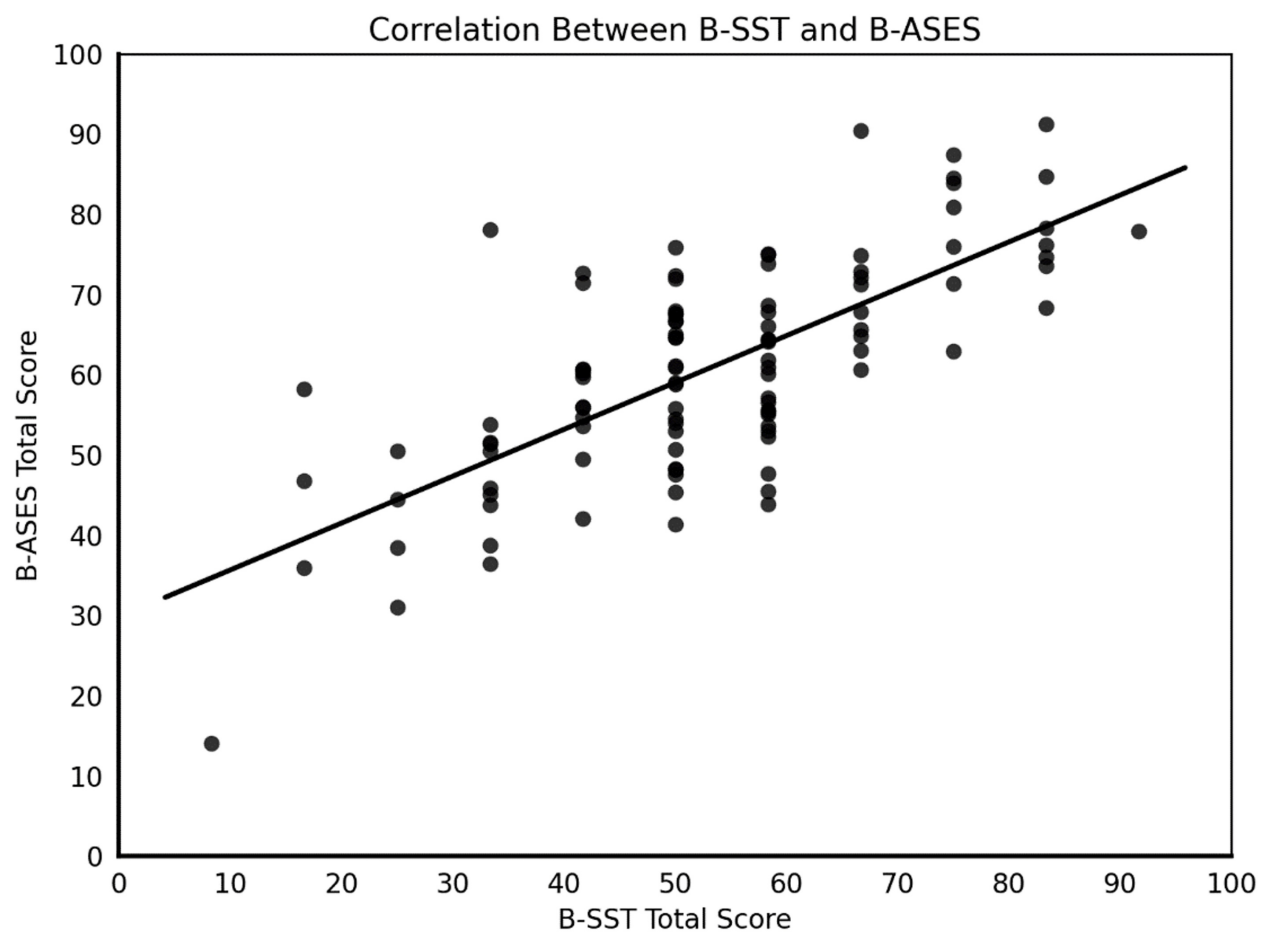
Convergent validity between the Bulgarian Simple Shoulder Test (BSST) and Bulgarian American Shoulder and Elbow Surgeons Shoulder Assessment Form (BASES) Pearson r = 0.71, p < 0.0001

A strong level of convergent validity was demonstrated between BSST and BASES, with a Pearson correlation coefficient of 0.719 (p < 0.0001) (Figure 1). Floor and ceiling effects were assessed using the 10th and 90th percentile cut-off points of the total possible score range. In the BSST, 0.97% of participants fell within the floor and ceiling score ranges, respectively. For the BASES, 1.94% of respondents reached the ceiling range, while no floor effect was identified. As all proportions remained well below the 15% criterion, no relevant floor or ceiling effects were observed, indicating an adequate distribution of scores. 

Reliability and Internal Consistency

All participants completed a second assessment 14 days following the initial evaluation. Test-retest reliability analysis revealed excellent agreement for both instruments, with intraclass correlation coefficients of 0.96 (95% CI 0.94-0.97, p < 0.0001) for the BSST and 0.93 (95% CI 0.91-0.96, p < 0.0001) for the BASES (Table 2). Evaluation of internal consistency showed that the BSST exhibited acceptable reliability (Cronbach’s α = 0.77), while the BASES demonstrated good internal consistency (Cronbach’s α = 0.86).

**Table 2:** Reliability of the BSST and BASES ICC: intraclass correlation coefficient, BSST: Bulgarian Simple Shoulder Test, BASES: Bulgarian American Shoulder and Elbow Surgeons Shoulder Assessment Form

Instrument	Baseline score	Test-retest score	Test-retest reliability	p-value
	mean±SD	mean±SD	ICC	
BSST (0-12 scale)	6.35±2.0	6.39±2.11	0.96, 95% CI 0.94-0.97	p < 0.0001
BSST (0-100 scale)	52.91±16.74	53.25±17.58		
BASES	60.74±13.59	60.77±14.83	0.93, 95% CI 0.91-0.96	p < 0.0001

Standard Error of Measurement and Minimal Detectable Change

The standard error of measurement (SEM) and the smallest detectable change at the 95% confidence level (SDC₉₅) were determined for both outcome measures. For the BSST, the SEM was 0.53, corresponding to an SDC₉₅ of 1.46 points. In the BASES, the SEM was calculated as 4.57, yielding an SDC₉₅ value of 12.66. These findings indicate satisfactory measurement accuracy and responsiveness. Accordingly, only changes exceeding 1.46 points on the BSST (scale range 0-12) and 12.66 points on the BASES can be considered to reflect a genuine change in patient status beyond measurement error.

Discriminant Validity

Discriminant validity was assessed by comparing BSST and BASES scores between a subgroup of 42 patients from the test-retest cohort (29 men and 13 women; mean age 46.4 ± 4.7 years) and a control group of 42 individuals without shoulder disorders (25 men and 17 women; mean age 39.0 ± 6.2 years). The demographic and clinical characteristics of both groups are summarized in Table 3. Independent-samples t-test analysis demonstrated statistically significant differences in BSST and BASES scores between patients and healthy controls.

**Table 3:** Scores of patients from the test-retest group and healthy controls (n = 42) BSST: Bulgarian Simple Shoulder Test, BASES: Bulgarian American Shoulder and Elbow Surgeons Shoulder Assessment Form

Instrument	Mean ± SD		Minimum-maximum		p-value
	Patients	Controls	Patients	Controls	
BSST (0-12 scale)	6,49±2.03	11.7±0.4	1.0-12.0	10.0-12.0	t(82)= –21.42 p<0.0001
BSST (0-100 scale)	54.08±16.91	97.5±3.33	8.33-100	83.33-100	
BASES	61.12±13.83	98.4±2.5	7.6-100.0	88.3-100.0	t(82)= –17.41 p<0.0001

## Discussion

Most self-administered outcome measures are originally developed in English, necessitating accurate translation and cultural adaptation before they can be applied in non-English-speaking populations. In line with previously published validation studies, the Bulgarian adaptation of the SST and ASES followed a structured forward-backward translation process, with cultural modifications implemented to ensure relevance to the Bulgarian context. Imperial measurement units, such as pounds and yards, were converted to metric equivalents commonly used in Bulgaria, consistent with adaptations performed in other language versions, including Thai and Persian [10, 16].

Both instruments demonstrated excellent test-retest reliability, with ICC values of 0.96 for the BSST and 0.93 for the BASES. Internal consistency was found to be acceptable to good, as reflected by Cronbach’s alpha coefficients of 0.77 and 0.86, respectively. In addition, discriminant validity was supported by the ability of both questionnaires to clearly differentiate between patients with shoulder disorders and asymptomatic individuals.

The SST offers a brief and user-friendly approach to assessing shoulder function, typically requiring less than three minutes to complete, making it particularly suitable for routine outpatient use. Previous studies have shown strong correlations between the SST and other shoulder-specific instruments, supporting its applicability across a broad spectrum of shoulder conditions, including instability, rotator cuff tears, and adhesive capsulitis [17, 18]. Comparable reliability has been reported in multiple translated versions of the SST, including the Japanese, Arabic, Persian, Brazilian, and Thai adaptations, with ICC values ranging from 0.61 to 0.95, which is consistent with the findings of the present study [16-19]. Notably, the Persian SST was validated concurrently with another PROM, the Oxford Shoulder Score, following a methodology similar to that used in the current investigation [16].

In contrast, fewer validated translations of the ASES shoulder form are available, with published versions including Portuguese, Italian, Arabic, and Turkish adaptations [20-23]. The Arabic and Turkish versions demonstrated psychometric properties comparable to those observed in the present study, reporting Cronbach’s alpha values of 0.81 and 0.88 and ICCs of 0.96 and 0.94, respectively. The psychometric performance of the ASES has also been evaluated in diverse clinical settings and across a wide range of shoulder conditions, both preoperatively and postoperatively [24-26]. Michener et al. assessed the measurement properties of the ASES in a cohort of sixty-three patients with various shoulder diagnoses, reporting lower test-retest reliability (ICC = 0.84) but identical internal consistency (Cronbach’s alpha = 0.86) compared with the present findings [12]. Overall, the BSST and BASES demonstrated consistent reproducibility across different shoulder pathologies, in agreement with previously published validation studies.

Several limitations should be acknowledged. First, construct validity was assessed by comparing two newly translated instruments rather than against an already validated Bulgarian measure, which may limit the strength of conclusions regarding construct validity, although this approach is consistent with hypothesis-testing methods and has been used in previous cross-cultural validation studies [16]. Second, clinical stability during the 14-day test-retest interval was not formally assessed using instruments such as the Global Rating of Change scale, which may have influenced reliability estimates. Third, structural validity was not evaluated using methods such as confirmatory factor analysis [27]. Although the SST and ASES are generally considered to be unidimensional instruments and similar validation studies have not consistently included factor analysis, further research may be warranted to confirm the dimensional structure of the Bulgarian versions [10, 20]. Fourth, a difference in age between the patient and control groups may have acted as a confounding factor in the assessment of discriminant validity, although the observed differences in scores were substantial. Finally, responsiveness was not assessed, as no therapeutic intervention was applied during the study period, and future studies should evaluate sensitivity to clinical change.

## Conclusions

In conclusion, the SST and ASES shoulder-specific questionnaires were successfully translated and culturally adapted into Bulgarian using established methodological standards. The results indicate that the Bulgarian versions of both instruments demonstrate good reliability, internal consistency, and evidence of convergent validity.

These tools are appropriate for evaluating shoulder pain and functional impairment in Bulgarian-speaking patients and may be used to monitor treatment outcomes. The availability of these instruments provides a valuable basis for future clinical and research work in shoulder pathology within Bulgarian-speaking populations.

## Appendices

Appendix A 

**Table 4:** Bulgarian Simple Shoulder Test The scale has been translated from the source article, and permissions to use and translate have been taken [6].

Прост тест за рамо		
	Да	Не
1. Удобно ли е на рамото ви, когато ръката ви е в покой до тялото?		
2. Рамото ви позволява ли ви да спите спокойно?		
3. Можете ли да се пресегнете до кръста си, за да подпъхнете ризата си в панталоните?		
4. Можете ли да поставите ръката си зад главата с изпънат настрани лакът?		
5. Можете ли да поставите монета върху рафт на нивото на рамото ви, без да сгъвате лакътя?		
6. Можете ли да повдигнете половин килограм (бутилка с вода от 500 мл) до нивото на рамото ви, без да сгъвате лакътя?		
7. Можете ли да повдигнете около 4 кг (две двулитрови бутилки) до нивото на рамото ви, без да сгъвате лакътя?		
8. Можете ли да носите тежест около 10 кг със засегнатата ръка?		
9. Смятате ли, че можете да хвърлите топка за тенис на корт отдолу и напред на около 20 метра със засегнатата ръка?		
10. Смятате ли, че можете да хвърлите топка за тенис на корт през рамо на около 20 метра със засегнатата ръка?		
11. Можете ли да измиете гърба на здравото си рамо със засегнатата ръка?		
12. Рамото ви ще позволи ли да вършите обичайната си работа на пълен работен ден?		

Appendix B 

**Table 5:** Bulgarian American Shoulder and Elbow Surgeons Shoulder Assessment Form The scale has been translated from the source article, and permissions to use and translate have been taken [11].

Българска версия за оценка по скалата на „Американските раменни и лакътни хирурзи“															
Обичайна работа															
Обичайна спортна/развлекателна дейност															
Имате ли болка в рамото през нощта?	Да						Не								
Приемате ли обезболяващи като парацетамол (ацетаминофен), диклофенак или ибупрофен?	Да						Не								
Приемате ли силни обезболяващи като кодеин, трамадол или морфин?	Да						Не								
Колко хапчета приемате средно на ден?															
Сила на болката? (оградете едно)	0	1		2	3	4		5		6	7	8		9	10
Брой точки	+0		+1						+2				+3		
Трудно ли ви е да облечете връхна дреха?	Не мога		Много трудно						Донякъде трудно				Не е трудно		
Трудно ли ви е да спите на болната страна?	Не мога		Много трудно						Донякъде трудно				Не е трудно		
Трудно ли ви е да си измиете гърба/ да си сложите сутиен?	Не мога		Много трудно						Донякъде трудно				Не е трудно		
Трудно ли ви е да се обслужвате в тоалетната?	Не мога		Много трудно						Донякъде трудно				Не е трудно		
Трудно ли ви е да сресвате косата си?	Не мога		Много трудно						Донякъде трудно				Не е трудно		
Трудно ли ви е да достигнете висока етажерка?	Не мога		Много трудно						Донякъде трудно				Не е трудно		
Трудно ли ви е да вдигнете 4.5 кг над рамото си?	Не мога		Много трудно						Донякъде трудно				Не е трудно		
Трудно ли ви е да хвърлите топка през рамо?	Не мога		Много трудно						Донякъде трудно				Не е трудно		
Трудно ли ви е да вършите обичайната си работа?	Не мога		Много трудно						Донякъде трудно				Не е трудно		
Трудно ли ви е да извършвате обичайната спортна/ развлекателна дейност?	Не мога		Много трудно						Донякъде трудно				Не е трудно		
Точкова оценка указания: Стойност на въпрос 7: _______ точки Резултат за болка: 5 x (10 - стойност на въпрос 7) Оценка за болка: _______ точки Въпросник за ежедневните дейности (ВЕД): Суров резултат за ВЕД: _______ точки Резултат за ВЕД: 5 x (суров резултат) / 3 Оценка за ВЕД: _______ точки Крайна оценка по скалата на ASES: оценка за болка + оценка за ВЕД Крайна оценка _______ точки															
